# Direct Nanoparticle Sensing in Liquids with Free-Space Excited Optical Whispering-Gallery-Mode Microresonators

**DOI:** 10.3390/s25165111

**Published:** 2025-08-18

**Authors:** Davide D’Ambrosio, Saverio Avino, Gianluca Gagliardi

**Affiliations:** Consiglio Nazionale delle Ricerche, Istituto Nazionale di Ottica (INO), Via Campi Flegrei, 34-Comprensorio A. Olivetti, 80078 Pozzuoli, Italy; saverio.avino@ino.it (S.A.); gianluca.gagliardi@ino.cnr.it (G.G.)

**Keywords:** whispering gallery modes, optical microresonator, imaging, nanoparticles, LorenzMie scattering, free-space light coupling

## Abstract

Whispering-gallery-mode (WGM) microresonators are amongst the most promising optical sensors for detecting bio-chemical targets. A number of laser interrogation methods have been proposed and demonstrated over the last decade, based on scattering and absorption losses or resonance splitting and shift, harnessing the high-quality factor and ultra-small volume of WGMs. Actually, regardless of the sensitivity enhancement, their practical sensing operation may be hampered by the complexity of coupling devices as well as the signalprocessing required to extract the WGM response. Here, we use a silica microsphere immersed in an aqueous environment and efficiently excite optical WGMs with a free-space visible laser, thus collecting the relevant information from the transmitted and back-scattered light without any optical coupler, fiber, or waveguide. We show that a 640-nm diode laser, actively frequency-locked on resonance, provides real-time, fast sensing of dielectric nanoparticles approaching the surface with direct analog readout. Thanks to our illumination scheme, the sensor can be kept in water and operate for days without degradation or loss of sensitivity. Diverse noise contributions are carefully considered and quantified in our system, showing a minimum detectable particle size below 1 nm essentially limited by the residual laser microcavity jitter. Further analysis reveals that the inherent laserfrequency instability in the short, -mid-term operation regime sets an ultimate bound of 0.3 nm. Based on this work, we envisage the possibility to extend our method in view of developing new viable approaches for detection of nanoplastics in natural water without resorting to complex chemical laboratory methods.

## 1. Introduction

In recent years, optical microresonators have attracted a considerable interest for sensing applications and have been developed with different geometries and dielectric materials [1,2,3,4]. They exhibit optical whispering gallery modes (WGMs) [3], low-loss electromagnetic waves guided by multiple total internal reflections along equatorial trajectories near the cavity surface. The prominent features of WGM microresonators, including low surface roughness and high-quality factors Q (>10^9^), imply an ultra-long intracavity photon lifetime and a strong light-field confinement, making them extremely sensitive to refractive index changes and absorption of the outer medium as well as surface scattering [5,6]. As an example, silica microspheres were used for detection of proteins and viruses measuring WGM shifts caused by corresponding binding events [7,8]. Also, interferometric techniques were adopted to detect nanoparticles [9], while plasmonic nanoparticle interaction was harnessed to increase the intrinsic sensitivity for biosensing [10]. Indeed, the interaction of WGMs with plasmonic nanoparticles can lead to a significant enhancement of the local radiation field that amplifies the refractometric and opto-thermal effects of the targets. Nevertheless, this sensitivity gain comes at the cost of increased complexity and does not always correspond to improvement in the overall detection limits. In fact, the lowest detectable sensor signal is fundamentally set by technical and environmental noise, unless the system is limited by pure electronic or quantum noise, which is rarely the case. Last but not least, the majority of WGM biosensors reported to date rely on prism-based or taperedfiber optical couplers. On one hand, tapered fibers are prone to deterioration due to both their intrinsic fragility and their susceptibility to environmental pollution. On the other hand, prism-based solutions are more robust, but typically require highly customized cells to be used in aqueousenvironment based experiments [2,11]. Hence, in most WGM-based sensors, the detection process may be seriously affected by fluctuations of the interrogating and readout setup, i.e., laser frequency and amplitude noise, as well as instabilities and spurious optical signals due to ambient fluctuations as well as coupling and collecting devices [12].

Compared to mature and widely commercialized sensing schemes such as surface plasmon resonance (SPR) [13], and single-particle counting techniques, including flow cytometry, nanoparticle tracking analysis (NTA), and digital ELISA [14], WGM sensors offer potentially higher sensitivity thanks to stronger light–matter interaction and reduced complexity without resorting to labeling methods. Indeed, even if the existing single-particle sensing modalities enable discrete detection with excellent specificity and resolution, they often require fluorescent or magnetic labeling.

Here, we developed a scheme based on a homemade silica microsphere directly immersed in liquid water. The microcavity is optically excited by a free-space laser beam focused tangential to its edge [15]: this gives rise to high-quality factor whispering-gallery light waves exhibiting an evanescent-wave tail that leaks out in the surrounding medium. The absorbtion of compounds or nanoparticles can be detected by the evanescent light field with maximum sensitivity when the laser is actively locked on the microsphere resonance with a high-bandwidth servo control. Thus, no post-processing is needed to detect and quantify the analytes, as the analog control signal provides all the desired information in real time and a wide bandwidth.

## 2. Materials and Methods

**Figure 1:**
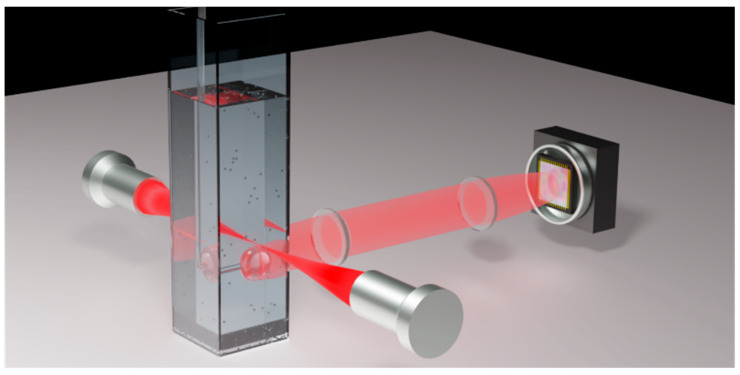
Experimental setup. A silica microsphere resonator is immersed in a liquid cuvette with transparent windows to excite WGMs by an edge-focused free-space laser beam. Objective lenses are employed to collect light scattered from the microsphere equator for particle imaging on a high-resolution camera.

Figure 1 displays a 3D representation of the optical setup’s primary components. A spheroidal bulge, fabricated through controlled fusion of the tip of a silica single-mode optical fiber, is used as a microresonator. The fiber stem is used for manipulation and alignment. The interrogation laser is a distributed feedback (DFB) diode laser with an emission wavelength of about 640 nm and a linewidth of 10 MHz. The laser beam is coupled to WGM resonances through free-space Lorenz–Mie scattering [16]. To this aim, a microscope objective focuses the light beam tangentially to the microsphere rim. In this way, an angular momentum (AM) matching condition is fulfilled for WGMs with j ≈ 2πd/λ, where j is the eigenvalue of the AM-squared operator. Upon illumination, we collect both the transmitted and scattered beams. The former is sent to a fast photodiode, while the latter is imaged through a 1:1 telescope on a CCD camera.

**Figure 2:**
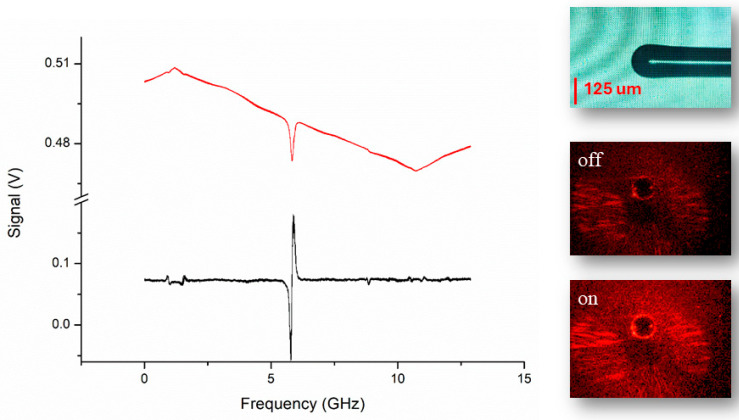
The interrogation laser wavelength is swept across a WGM resonance (red trace) of a spherical resonator (R ≈ 70 µm, upper right panel). The FWHM is about 150 MHz, thus showing a Q-factor of 3 × 10^6^. The black trace is the PDH error signal used to lock the laser frequency to the cavity resonance. In the lower right panels are the images collected by the CCD camera when the laser is on and off resonance.

Our microresonators have typical diameters ranging from 100 µm to 200 µm, and are immersed in a glass cuvette containaing MilliQ^®^water (Sigma-Aldrich, Merck KGaA, Darmstadt, Germany), leveraging the versatility of free-space WGM coupling. WGMs can readily be observed when the diode laser wavelength is swept across a resonance condition (Figure 2). If a nanoparticle diffuses through the liquid buffer and reaches the microsphere’s surface, the WGM experiences a reactive shift proportional to the nanoparticle’s dimension and polarizability [1]. The laser frequency is locked to the WGM resonance by means of a Pound-Drever-Hall scheme to track any particle-induced WGM shift with maximum sensitivity and minimum response time [2]. For this purpose, the diode laser is phase modulated by a radiofrequency (RF) sinusoidal wave sent to its injection current to generate optical sidebands. Demodulation of the cavity-transmitted power with an RF mixer yields a dispersive-like, zero-crossing error signal (Figure 2, black trace). The error signal is sent to a proportionalintegrative custom-built electronic servo that generates the feedback voltage necessary to keep the laser locked to the resonance peak within a 1-kHz bandwidth.

In this way, any optical-frequency shift due to nanoparticle adsorption is translated by the servo in a voltage proportional to this shift, i.e., to the nanoparticle’s dimension, thus providing a direct, real-time readout with a large dynamic range and high bandwidth. To calibrate the overall system response, we vary the laser current in an open-loop configuration and measure the corresponding resonance shift to determine the tuning parameter in mA/kHz. Next, we activate the locking loop and observe the PDH correction signal response to a known external modulation, thereby extracting the calibration coefficient in mV/mA. By combining these two measurements, we derive a final transfer function expressed in nV/kHz. The resonance shift was transformed into laser frequency detuning using a fixed Fabry–Perot interferometer as a ruler to calibrate the WGM spectra.

## 3. Results

Three groups of silica nanoparticles, with nominal sizes of 300, 100, and 20 nm (relative standard deviation RSD ≤ 12%), were selected as the targets for the detection experiments.

**Figure 3:**
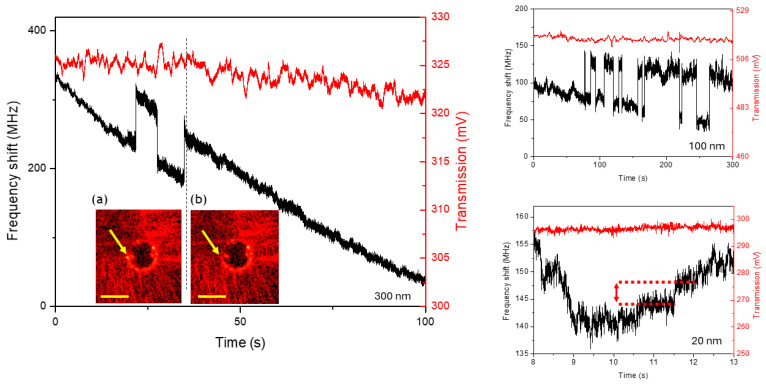
Silica nanoparticles in water interacting with WGMs. In red, the transmitted light power detected by a fast photodiode; in black, the corresponding correction signal to the laser. Landing events of nanoparticles with a nominal diameter of 300 nm: the inset images show a particle just before (**a**) and after (**b**) detaching from the surface, thus generating a step on the feedback signal (yellow bars corresponding to 140 µm). Right-upper panel, analogous signals observed for 100 nm nanoparticles. Right-lower panel, small signals for 20 nm nanoparticles are also observed.

In Figure 3, we show the WGM shifts induced by different silica nanoparticles dispersed in the water cuvette (concentration of 1 µg/mL) where the 70-µm microresonator is immersed. The shifts manifest as small negative steps in the locking feedback (correction) signal while the transmission remains reasonably stable on a timescale of a few minutes. A small slope on the transmission line may occasionally be present due to thermal drifts in the WGM that are fed back to the laser injection current to keep it locked. On the left-hand side of Figure 3, subsequent steps are seen as a single 300-nm nanoparticle lands on the microsphere’s surface, which then takes off and lands again. A similar transition can also be appreciated in the inset images where the microresonator’s equator exhibits a bright spot (a) that turns off (b) after detachment. Moreover, the step height quantifies the energy spent by the optical evanescent field when a target interacts with the WGM, thus enabling nanoparticle sizing. Using the formula given in [1], we can retrieve the nanoparticle size from the observed WGM shift. The correction signal provides a direct measurement of this shift, and thus of the diameter, which turns out to be 480 nm in this first case, and proves quite stable in subsequent adsorption events. The discrepancy found between the measured value and the nominal size is possibly due to a difference between the open-loop and the closed-loop response, which affects the actual wavelength-shift calibration. Analogously, Figure 3’s upper-right graph shows attachment and detachment events when the microsphere is immersed in a 100 nm nanoparticle suspension, with the correction signal yielding, in this case, a measured diameter of about 350 nm. Finally, in Figure 3’s lower-right graph, we show the recording for 20 nm nanoparticles in water. Al measured values are summarized in Table 1.

**Figure 4:**
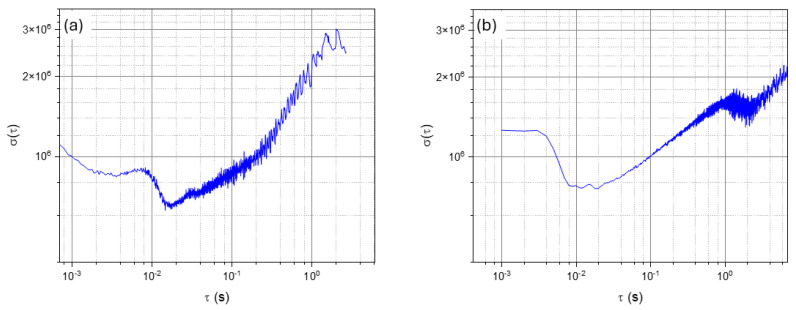
Allan deviation of the (**a**) output of the WGM locking servo (correction), (**b**) laser stability from the side-of-fringe measurement with a Fabry-Pérot interferometer.

In order to estimate the current detection limit, we analyzed the correction signal, calibrated in terms of optical frequency shift, over a long acquisition. Thus, in Figure 4a, we calculated the Allan deviation in the experimental data taken without nanoparticles: for an observation time of 1 s, we obtained an apparent fluctuation of 3 MHz, i.e., a minimum detectable nanoparticle size of ~0.8 nm, which compares very well with the state of the art [17]. For longer timescales, the Allan deviation increases as thermal drifts dominate the measurement. As we will show in the following, this is not the least possible level for our WGM sensor.

A figure of merit of the ultimate detection capability of our system was extracted from the intrinsic laser frequency stability. As shown in Figure 4b, we characterized it using the side-of-resonance measurements of a stable FP external cavity scanning through the laser emission mode, yielding a stability of ~1 MHz on a 1-s timescale. The difference between the direct interrogation noise and that derived from the laser contribution may be likely due to the finite locking gain between the laser and the WGM that provides the real-time readout. For instance, the resonance FWHM is 150 MHz, and the extent to which the interrogating laser is tracking its center depends on the overall servo performance.

## 4. Conclusions

Nanoparticle detection is demonstrated using a homemade optical WGM microresonator operating in aqueous environments. The system relies on a visible laser that excites WGMs on a silica microsphere via free-space scattering without resorting to waveguides, prisms, or any coupling device, thus enabling direct sensing and imaging of the sensing region where the evanescent tail interacts with the target analytes landing on the surface. Reactive shift effects result in direct analogic readout thanks to the laser frequency locking to the resonance, which offers fast, reliable sensing also on a long timescale with detection limits below 1 nm, in terms of the minimum nanoparticle size. Further improvements can readily be obtained by replacing the interrogation source with a narrower emission or a frequency-stabilized laser. In this case, a higher Q factor would result in a narrower resonance, thus leading to a higher spectral resolution in reactive shift measurements. The system developed in our work paves the way to new schemes for the fast detection of dielectric nanoparticles in natural water samples. Thanks to the optical WGM reactive shift, which depends merely on the target’s size and permittivity [1], this approach shows high versatility and can be readily extended to nanoparticles made of different kinds of materials, with the most commonly used ones being TiO_2_, SiO_2_, Ag and Au, but also polymers, i.e., nanoplastics [18] The presence of nanoplastics in the environment is a serious public concern and optical sensing methods, after careful testing, could enable their detection and characterization without resorting to mass spectrometry or complex chemical laboratory methods.

## Figures and Tables

**Table 1:** Summary of expected and measured nanoparticle dimensions.

Nominal (nm)	300	100	20
Measured (nm)	480	350	90

## Data Availability

The data presented in this study are available on request.
